# Addressing complicated vesicovaginal fistula post-intercourse in MRKH: the critical imperative for comprehensive sex education and awareness

**DOI:** 10.1093/sexmed/qfae076

**Published:** 2026-03-12

**Authors:** Gautam Shubhankar, Ankur Mittal, Vikas Kumar Panwar, Deelip Kumar Singh

**Affiliations:** Urology, All India Institute of Medical Sciences (AIIMS), Rishikesh 249203, Uttarakhand, India; Urology, All India Institute of Medical Sciences (AIIMS), Rishikesh 249203, Uttarakhand, India; Urology, All India Institute of Medical Sciences (AIIMS), Rishikesh 249203, Uttarakhand, India; Urology, All India Institute of Medical Sciences (AIIMS), Rishikesh 249203, Uttarakhand, India

**Keywords:** MRKH, vesicovaginal fistula, post-intercourse, VVF

## Abstract

**Introduction:**

Mayer-Rokitansky-Küster-Hauser (MRKH) syndrome, a rare congenital disorder, affects approximately 1 in 4500 to 5000 females, leading to the underdevelopment or absence of the uterus and upper vaginal segment. While the physical manifestations of MRKH are substantial, the associated psychological and social repercussions, exacerbated by societal norms, can be profound. Inadequate sex education further compounds the challenges faced by these women, particularly in postmarital contexts. This report highlights an unprecedented case of a young woman with MRKH type II who developed a complicated vesicovaginal fistula (VVF) following vaginal intercourse, underscoring the urgent need for enhanced sex education and awareness.

**Aims:**

This study aims to document and analyze a unique case of post-coital VVF in a patient with MRKH syndrome, illustrating the consequences of inadequate sex education. The broader objective is to advocate for comprehensive sexual and reproductive health education, particularly in regions with limited access, to prevent such complications and improve the quality of life for affected individuals.

**Methods:**

A detailed case study of a young woman in her early twenties with MRKH type II is presented. The patient experienced continuous urine leakage per vaginum following intercourse, which was preceded by dyspareunia and bleeding. A clinical examination revealed a large, complicated VVF involving the bladder neck and posterior bladder wall, coupled with a left ectopic kidney as confirmed by ultrasound. The patient received broadspectrum antibiotics, wound care, and extensive psychosocial counseling, with an emphasis on sex education for her and her family. A multidisciplinary approach was employed, planning delayed VVF repair and vaginoplasty.

**Results:**

The patient’s wound gradually healed with conservative management, and her psychosocial outlook improved significantly following counseling and education. The family’s understanding of MRKH and its implications was enhanced, reducing stigma and promoting a supportive environment. Surgical planning for reconstructive procedures was successfully initiated, with the patient showing positive engagement in her care.

**Conclusion:**

This case highlights the critical importance of comprehensive sex education for individuals with MRKH syndrome, particularly in underserved regions. The development of a complex VVF post-intercourse is a stark reminder of the preventable complications arising from inadequate knowledge and awareness. Enhanced educational initiatives are imperative to equip patients and families with the understanding needed to mitigate risks, reduce stigma, and improve the overall wellbeing of those affected by MRKH. This case underscores the need for an integrated approach combining education, psychological support, and medical care to optimize outcomes for patients with MRKH.

## Introduction

Mayer-Rokitansky-Küster-Hauser (MRKH) syndrome is a congenital disorder affecting approximately 1 in 4500 to 5000 females, characterized by the underdevelopment or complete absence of the uterus and upper segment of the vagina.^1^ In MRKH type II, additional systemic features may include renal anomalies, such as ectopic or absent kidneys, and skeletal malformations, particularly vertebral or rib deformities. Less commonly, patients may present with auditory defects or congenital cardiac anomalies, necessitating multidisciplinary management to address the full spectrum of associated complications.

The syndrome typically occurs during adolescence and is often triggered by the absence of menstruation (primary amenorrhoea), which prompts medical investigation. Girls with MRKH generally develop normal secondary sexual characteristics such as breast development and pubic hair, since ovarian function remains intact. However, when menstruation fails to begin, usually between the ages of 15 and 17, further evaluation may reveal the characteristic underdevelopment or absence of the uterus and upper part of the vagina.

The diagnosis can be particularly distressing for adolescents, as it coincides with a period of significant emotional and social development, where peer relationships and societal expectations related to femininity and future motherhood are prominent. This sudden discovery often leads to feelings of shock, confusion, and inadequacy, as many girls and their families may be unaware of the condition until this pivotal moment. The realization not only disrupts the adolescent’s emerging identity but also brings about long-term implications related to fertility and sexual health, heightening the need for early counseling and support to mitigate psychological distress.

The long-term sequelae of MRKH syndrome extend far beyond its initial diagnosis, deeply affecting multiple facets of an individual’s life. Infertility, one of the most profound consequences, stems from the absence of a functional uterus, though ovarian function is typically normal, leading to emotional challenges regarding motherhood. Options such as surrogacy or adoption present alternatives, but they carry their own psychological and societal pressures. Psychosexual difficulties also arise due to the absence of a functional vagina, often necessitating surgical or nonsurgical interventions like vaginoplasty or dilation therapy, further compounding body image concerns and sexual dissatisfaction. The psychological toll is immense, with many patients experiencing anxiety, depression, and feelings of inadequacy due to societal expectations of menstruation and motherhood. MRKH type II can also bring chronic health challenges, such as renal or skeletal anomalies, requiring ongoing monitoring and medical care. Additionally, delayed diagnosis—often due to limited awareness—can heighten the emotional burden, as early intervention is crucial in alleviating both physical and mental distress. Thus, a comprehensive, multidisciplinary approach is essential for improving long-term outcomes and quality of life.^2^

In light of these challenges, we encountered a troubling case of a young woman in her early twenties with MRKH type II who presented with a complex vesicovaginal fistula (VVF) as a consequence of vaginal intercourse. This case represents the first documented instance of a coital injury in a patient with MRKH syndrome resulting in a complicated VVF. The case starkly illustrates the critical need for improved sex education and awareness, as it highlights the preventable complications arising from insufficient knowledge and understanding of MRKH.

## Methods

**Figure 1:**
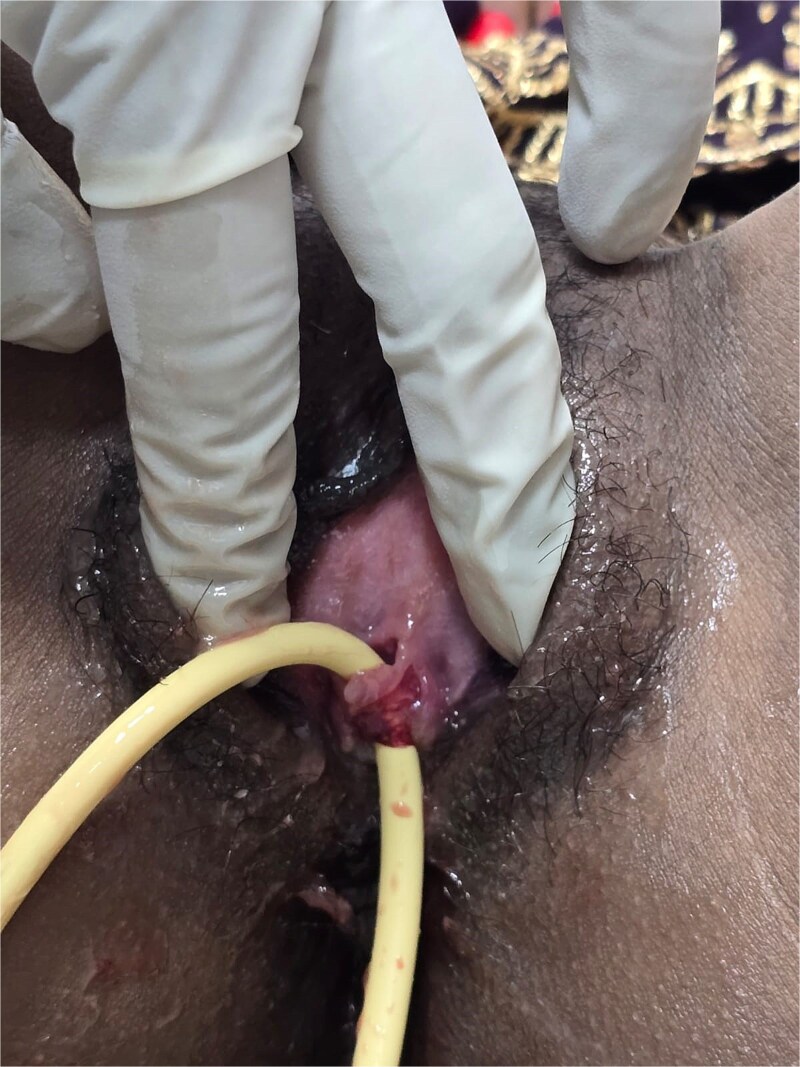
Showing Foleys catheter inside the urethra only for the initial 2 cm beyond which it came out through the complicated vesicovaginal fistula extending till the bladder neck.

**Figure 2:**
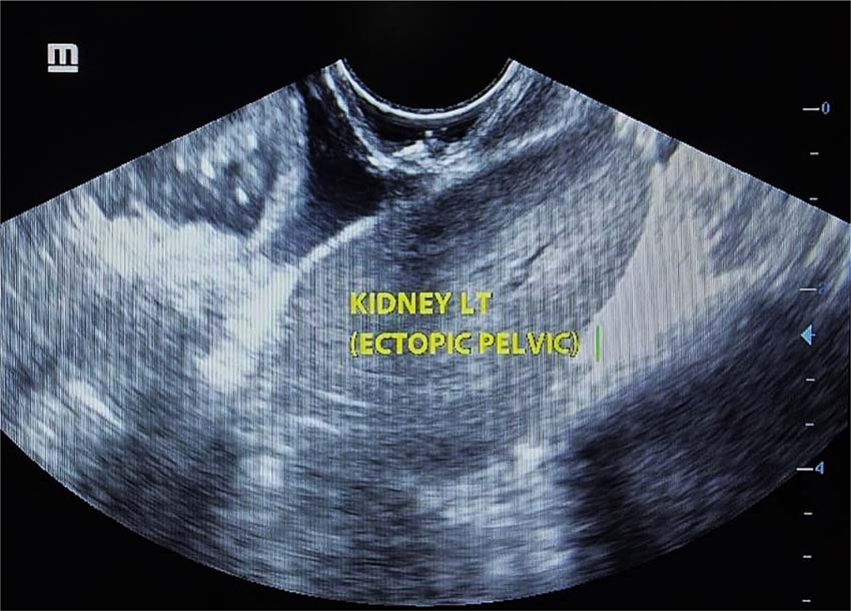
Ultrasound of abdomen showing left ectopic pelvic kidney.

A young female in her early twenties presented with continuous urine leak per vaginum post-repeated attempts of vaginal intercourse after marriage. The event was preceded by dyspareunia and bleeding per vaginum. She had primary amenorrhoea with normal secondary sexual characteristics with examination revealing a “bind-ending” vagina that was torn as a result of repeated attempts of vaginal penetration, finally giving rise to a large 5 × 4 cm complicated VVF involving the bladder neck and the posterior wall of the urinary bladder. An attempt at Foley catheter insertion revealed that the catheter could be tubed only in the initial 2 cm of the urethra (Figure 1). Beyond that, it opened into an open vesicovaginal space (bounded anteriorly by the anterior bladder wall and posteriorly by the posterior vaginal wall) created as a result of the ruptured posterior bladder wall and anterior wall of the bind-ending vagina. Ultrasound of the abdomen revealed a left ectopic kidney thus labeling her as MRKH type II (Figure 2).

## Result

The patient was admitted and was started on broad-spectrum antibiotics (Injection Meropenem and Amikacin). Her urine culture also revealed Escherichia coli, which was sensitive to Meropenem and Amikacin. Daily cleaning and dressing of the vaginal wound was done and she was advised for sitz bath daily to ensure proper local hygiene. Multiple counseling sessions were done for the patient, her parents, and her husband regarding the syndrome she has been harboring since birth and the current circumstances as a result of intercourse. The patient underwent comprehensive counseling over several weeks, with sessions conducted twice a week. These sessions involved a multidisciplinary approach, including a gynecologist, psychologist, and sexual health educator to ensure a holistic understanding of the condition.

Initially, the counseling focused on educating the patient and her family about MRKH, including its anatomical and reproductive implications. This stage also addressed the emotional burden of the diagnosis, helping the patient and her family come to terms with the reality of primary amenorrhoea and the associated fertility challenges. Subsequent sessions concentrated on sexual health education, particularly the nature of the vaginal abnormality and safe sexual practices to prevent further complications. Psychological support was ongoing throughout the sessions, aimed at alleviating feelings of inadequacy and social stigma while promoting body positivity and emotional resilience.

Importantly, the husband and family were actively involved in the sessions to foster a supportive environment, reduce societal pressures, and facilitate open communication about the patient’s condition. By providing consistent and empathetic counseling over an extended period, the healthcare team ensured that the patient could not only process the diagnosis but also strengthen her emotional well-being, prepare for potential surgical interventions, and improve her relationship dynamics.

A thorough sex education and awareness were provided to them with proper feedback to filter out all the social stigma percolating in their minds. It took time, but gradually, they understood the situation with a positive mindset. Gradually her wound healed and psychosocially she became stronger to deal with the circumstances. She was advised to abstain from any coital activity, which the couple understood. Currently, she had been planned for bladder neck reconstruction with a delayed repair of the complicated VVF along with vaginoplasty.

## Discussion

The case of a young woman with MRKH syndrome who developed a complex VVF post-coital injury provides a critical insight into the profound implications of inadequate sex education and counseling. MRKH syndrome, a rare congenital disorder affecting 1 in 4500 to 5000 females, is characterized by the absence or underdevelopment of the uterus and upper part of the vagina, with MRKH type II presenting additional anomalies. The physical challenges associated with MRKH are significant, but the psychological and social repercussions are equally profound, with patients often facing substantial stigma related to societal expectations of femininity and procreation.

The Guttmacher Institute’s 2017 report highlights a stark reality: approximately 60% of adolescent girls in sub-Saharan Africa and South Asia lack access to comprehensive sexual and reproductive health services, including essential sex education. This educational gap severely impairs these young women’s ability to make informed decisions about their sexual and reproductive health, thus heightening their risk of preventable complications. The presented case, in which a young MRKH patient developed a VVF as a result of coital injury, underscores the critical need for comprehensive sex education. This complication, while rare, is a direct consequence of insufficient knowledge and understanding of MRKH and its implications.^3^

Vosoughi et al. demonstrated that targeted psychosexual education significantly enhances sexual function, genital self-image, and reduces sexual distress in women with MRKH. This evidence reinforces the need for systematic educational interventions to address both the medical and psychosocial aspects of MRKH.^4^ Effective management of MRKH and its complications extends beyond medical treatment to include robust educational and counseling support. Such interventions should encompass detailed education about the condition, its management, and preventative strategies to mitigate risks, especially in regions with lower literacy and educational standards.

In areas where educational resources are limited, it is crucial to implement community-based programs that provide accurate information and support. By addressing the educational deficit, healthcare providers can reduce the stigma associated with MRKH, empower patients, and prevent complications such as those illustrated in this case. A comprehensive approach integrating education, psychological support, and appropriate medical care is essential for improving outcomes and enhancing the quality of life for individuals with MRKH.

## Conclusion

This case underscores the critical need for comprehensive sex education and counseling for individuals with MRKH syndrome, particularly in underserved regions. The development of a complex VVF following coital injury highlights the significant consequences of inadequate knowledge about MRKH and its implications. Enhanced educational interventions and community-based programs are essential to equip patients and their families with the necessary understanding and support. By addressing these educational deficits, healthcare providers can mitigate complications, reduce stigma, and improve the overall quality of life for individuals affected by MRKH.
